# Ionic Liquids toward Enhanced Carotenoid Extraction from Bacterial Biomass

**DOI:** 10.3390/molecules29174132

**Published:** 2024-08-30

**Authors:** Tiago P. Silva, Luís Alves, Francisco Salgado, José C. Roseiro, Rafał M. Łukasik, Susana M. Paixão

**Affiliations:** 1 LNEG—Laboratório Nacional de Energia e Geologia, IP, Unidade de Bioenergia e Biorrefinarias, Estrada do Paço do Lumiar, 22, 1649-038 Lisboa, Portugalrafal.lukasik@lukasiewicz.gov.pl (R.M.Ł.); 2 Łukasiewicz Centre, Łukasiewicz Research Network, Research and Innovation Department, 19 Poleczki str., 02-822 Warsaw, Poland

**Keywords:** *Gordonia alkanivorans* strain 1B, carotenoids, extraction methodology, ionic liquids, green approach

## Abstract

Carotenoids are high added-value products primarily known for their intense coloration and high antioxidant activity. They can be extracted from a variety of natural sources, such as plants, animals, microalgae, yeasts, and bacteria. *Gordonia alkanivorans* strain 1B is a bacterium recognized as a hyper-pigment producer. However, due to its adaptations to its natural habitat, hydrocarbon-contaminated soils, strain 1B is resistant to different organic solvents, making carotenoid extraction through conventional methods more laborious and inefficient. Ionic liquids (ILs) have been abundantly shown to increase carotenoid extraction in plants, microalgae, and yeast; however, there is limited information regarding bacterial carotenoid extraction, especially for the *Gordonia* genus. Therefore, the main goal of this study was to evaluate the potential of ILs to mediate bacterial carotenoid extraction and develop a method to achieve higher yields with fewer pre-processing steps. In this context, an initial screening was performed with biomass of strain 1B and nineteen different ILs in various conditions, revealing that tributyl(ethyl)phosphonium diethyl phosphate (IL#18), combined with ethyl acetate (EAc) as a co-solvent, presented the highest level of carotenoid extraction. Afterward, to better understand the process and optimize the extraction results, two experimental designs were performed, varying the amounts of IL#18 and EAc used. These allowed the establishment of 50 µL of IL#18 with 1125 µL of EAc, for 400 µL of biomass (cell suspension with about 36 g/L), as the ideal conditions to achieve maximal carotenoid extraction. Compared to the conventional extraction method using DMSO, this novel procedure eliminates the need for biomass drying, reduces extraction temperatures from 50 °C to 22 ± 2 °C, and increases carotenoid extraction by 264%, allowing a near-complete recovery of carotenoids contained in the biomass. These results highlight the great potential of ILs for bacterial carotenoid extraction, increasing the process efficiency, while potentially reducing energy consumption, related costs, and emissions.

## 1. Introduction

Synthetic dyes/pigments have a long history of being used in different areas, from arts to textiles and even food/feed; however, consumer awareness on the environmental effects of the dyeing process, and potential toxic effects of the dyes themselves, has led the industries to search for sustainable and broadly available natural sources of coloration [1].

Carotenoids are a class of naturally synthesized lipid-soluble pigments that can have diverse coloration, ranging from pink and red to orange and yellow. As the name implies, these molecules are responsible for the colors of many plants, such as carrots or tomatoes, animals, such as salmon or shrimps, fungi, as well as several microorganisms, such as microalgae, yeasts, archaea, and some bacteria [2,3].

Carotenoids are regarded as high added-value products, with an estimated market of about USD 2.33 billion in 2032 [4], due to their diverse bioactive and chemical properties. Besides their intense coloration, they play crucial roles as essential nutrients, antioxidants with specific roles or general health-promoting roles that reduce the risk or progression of diseases associated with oxidative stress, absorbers of light energy, oxygen transporters, provitamin A, antitumor and enhancers of in vitro antibody production, antimicrobial agents, and structural components of microbial membranes [5,6,7,8,9,10,11]. Hence, there is a vast market for carotenoids in the industrialized world, where they are produced toward different industries, ranging from pharmaceutical, nutraceutical, food/feed additive, textile, cosmetics, to fine chemicals sectors.

Studies on microbial carotenoid production have been mostly centered on microalgae and yeast, but research on bacterial carotenoids has become an area of interest. Bacteria can produce several types of carotenoids, are easy to manipulate, have high growth rates, low nutritional demands, and can be cultivated in a variety of alternative carbon sources under a wide range of conditions, decreasing cultivation costs [11,12].

Nevertheless, despite these advantages, bacteria have lower carotenoid yields compared to other microorganisms, such as microalgae, making them less appealing toward commercial application. Therefore, different approaches have been proposed to increase carotenoid yield, such as manipulation of culture conditions (i.e., sequential nutrition starvation or induction of carotenoid accumulation in microbial cells by employing several stress factors) or genetic manipulation of bacterial strains to obtain hyper-carotenoid producers [13]. However, the lack of proper or optimized extraction methods can also contribute to the lower production yields, as most established carotenoid extraction processes were developed for plant cells and, more recently, microalgae and yeasts. Bacterial cells have very different properties, and many of the established protocols are sometimes unable to correctly assess the carotenoid content [13,14,15].

An important aspect that may hinder the industrial application of carotenoids is that conventional extraction methods typically depend on a combination of physical processing, such as cell rupturing, drying, or grinding, along with the abundant use of organic solvents. In fact, solvent extraction is one of the methods most used for carotenoid extraction [11,16]. However, these methods are both material and time-consuming, can lead to lower product yield due to carotenoid degradation, and present some health and environmental safety concerns [17]. This issue can be accentuated for bacteria isolated from extreme environments, such as hydrocarbon-contaminated soils, since they typically show adaptations (e.g., membrane composition; biosurfactant/bioemulsifier production) that make them more resistant to the action of organic solvents [18]. Hence, the search for novel, greener approaches that rely on less-conventional extraction techniques is of paramount importance to bypass these limitations, increasing the yields of carotenoids in shorter periods of time when compared to traditional techniques [11,19].

In this context, the use of ionic liquids (ILs) could prove to be an alternative to avoid many of the limitations of conventional extraction processes. Their properties vary depending on the cation and anion pair but, overall, ILs are salts, which, due to their bulky, unsymmetrical ions with a delocalized charge, are liquid < 100 °C, with many being liquid even at room temperature [20]. They can have high thermal and chemical stability, meaning they can be used in a wide range of temperatures and be conjugated with different compounds, while also having a large electrochemical window, great solvent power, non-flammability, and a negligible vapor pressure [21,22]. ILs can also facilitate chemical reactions, extraction and separation, and biotransformation, making them ideal solvents for multiple applications [23,24,25,26]. Given these characteristics and considering that most ILs are nonvolatile, recyclable, and nonexplosive, they are seen as more environmentally friendly alternatives when compared to conventional organic solvents.

Indeed, ILs have been successfully used for microbial carotenoid extraction as an environmentally friendly alternative for cell disruption and the consequent recovery of intracellular colorants. However, as with the conventional processes, most work has focused on microalgal and yeast cells, with far fewer references regarding bacterial carotenoid extraction [23,27].

Thus, the main goal of this study was to evaluate the potential of ionic liquids (ILs) to mediate bacterial carotenoid extraction and develop a new method to achieve higher yields with fewer pre-processing steps. In this context, the bacterium used for the assays was *Gordonia alkanivorans* strain 1B, which exhibits pink/orange pigmentation. It has been described as a hyper-pigment producer [28,29], with astaxanthin, lutein, and canthaxanthin as the main carotenoids identified. Moreover, this microorganism was originally isolated from oil-contaminated soils and, as such, its characteristics make it difficult to rupture the cells and perform carotenoid extraction through conventional methods. The method currently used is very laborious and time-consuming and has been reported to result in incomplete extraction, with biomass retaining much of its original coloration even after several extraction steps [28,29,30].

Several ILs are known for their ability to permeate cells and facilitate extraction of intracellular compounds, such as carotenoids. Therefore, many protocols combine an IL with other solvents [31,32,33,34], where the IL ruptures the cells and the co-solvent extracts the carotenoids, thus reducing the steps needed to attain effective extraction. However, depending on the characteristics of the cells, especially membrane composition, different types of ILs may be needed to achieve bacterial cell rupture.

Since there is little knowledge on how ILs influence bacterial carotenoid extraction, particularly for the genus *Gordonia*, the first step of this work was to perform a screening with different ILs with distinct properties and assess their effect on carotenoid extraction in combination with ethyl acetate (EAc), a solvent known to solubilize the carotenoids extracted from strain 1B. Additionally, their ability to be further separated from the extracted biomass and organic solvent was also evaluated. Once the IL that resulted in the highest carotenoid yield was selected, extraction conditions were then optimized using two sequential experimental designs, based on the Doehlert uniform shell design for two factors (volume of IL and volume of EAc), with the objective of better understanding the behavior of the selected IL and maximizing bacterial carotenoid extraction, while minimizing the utilization of both solvents (IL and EAc). After determining the best extraction conditions, the novel extraction procedure was further validated in comparison to the previous method described by Silva et al. [28].

## 2. Materials and Methods

### 2.1. Chemicals

Ethyl acetate (99.8%) was obtained from Carlo Erba (Emmendingen, Germany), dimethyl sulfoxide (DMSO) (99.7%) was from Sigma-Aldrich (Darmstadt, Germany), and all other reagents were of the highest grade commercially available. Ultrapure water from Purist Ultrapure Water Systems (18.2 MΩ, PURIST^®^, RephiLe Bioscience Ltd., Lisbon, Portugal) was used for all experiments. The list of ILs and their respective suppliers is shown in Table 1 (see Table S1 in the Supplementary Materials for the respective chemical structures).

### 2.2. Microorganism and Culture Conditions

The microorganism used in this study was the bacterium *Gordonia alkanivorans* strain 1B, isolated in our laboratory [35], and maintained as described by Silva et al. [36]. Bacterial biomass was cultivated, as described by Silva et al. [37], in a chemostat (about 700 mL working volume on a 3.3 L bench-top bioreactor, New Brunswick Scientific, Edison, NJ, USA), using a basal salts medium: 2.196 g/L NH_4_Cl, 1.0 g/L KH_2_PO_4_, 1.0 g/L Na_2_HPO_4_.2H_2_O, 0.0850 g/L MgCl_2_.6H_2_O, 0.065 g/L Na_2_SO_4_, and 0.250 mL/L of trace elements solution (TES) [38]. The bioreactor with the culture medium adjusted to a pH of 7.5 ± 0.2 was sterilized by autoclaving at 121 °C, 1 atm, for 30 min. Additionally, this culture medium was supplemented with a sterile C-source (fructose) for an initial concentration of 20 g/L. Moreover, sterile polypropylene glycol was also added at a concentration of 0.15 mL/L to control foam formation.

The chemostat was maintained at a dilution rate of about 0.07 h^−1^, at 30 °C, with a pH of 7.5 (maintained by additions of 2 M NaOH solution on demand), agitation of 425 rpm, and an aeration rate of 2 vvm (vol. of air/vol. liquid/min) for at least five turnovers of the culture medium, after which the culture was considered to be at steady state.

At steady-state, bacterial cells were collected to ice, and then centrifuged at 8600× *g* at 4 °C for 20 min (Sigma model 2-16K Centrifuge, Sartorius AG, Göttingen, Germany) to increase the biomass concentration, before being used in carotenoid extraction assays.

### 2.3. Carotenoid Extraction Using Ionic Liquids

#### 2.3.1. Initial Screening

Based on their chemical properties, several ILs were tested to optimize the methodology usually employed to extract the carotenoids produced by *G. alkanivorans* strain 1B, as previously described by Silva et al. [28] and Fernandes et al. [29]. Thus, the pigment extraction potential of 19 ILs (IL#1 to IL#19 described above in Table 1) with ethyl acetate as a co-solvent was evaluated.

Using 1.5 mL Eppendorf tubes, 400 µL of strain 1B wet biomass (50 gDCW/L, where DCW = dry cell weight), produced as described above, was mixed with each of the 19 ILs and EAc in a 4:3:3 ratio (i.e., 400 µL concentrated biomass suspension + 300 µL IL + 300 µL EAc), in a batch extraction assay, according to an adaptation of a method from Ruiz et al. [39]. After being homogenized in a vortex, the tubes were taken to an overhead shaker (Heidolph Reax 20, Schwabach, Germany) and left shaking overnight at room temperature (22 ± 2 °C). The samples were centrifuged at 15,000× *g* for 15 min (Biofuge 15 Centrifuge, Heraeus Sepatech, Hanau, Germany) to separate the biomass and extracted carotenoids.

Results were first evaluated qualitatively, based on visual confirmation of carotenoid extraction and clear separation of the extracted biomass, IL, and solvent containing the carotenoids. Conditions that allowed the separation of a layer containing the extracted carotenoids were then evaluated quantitatively through spectrophotometry, using a Shimadzu spectrophotometer UV-2401PC (Kyoto, Japan), in terms of total carotenoid extraction (µg/gDCW) from the wet biomass, as previously described [28,40].

#### 2.3.2. Experimental Design Methodology

According to the results obtained from the screening, IL#18 coupled with EAc was selected as the best procedure for further optimization. To minimize the number of optimization assays aimed at achieving high carotenoid extraction, a response surface methodology based on the Doehlert uniform shell design [41] for two factors (X_1_ = IL#18 volume and X_2_ = EAc volume) was used. Two sequential experimental designs (#ED1 and #ED2), with fourteen experiments each (seven conditions in duplicate), were performed:

- #ED1 had a wider range to broadly understand how each of the factors influenced the response. Thus, the volume of IL#18 (X_1_) ranged between 25 µL and 1000 µL, and the volume of EAc (X_2_) ranged between 250 µL and 2000 µL.
- #ED2 was based on the results of #ED1, with the goal of improving extraction efficiency. Therefore, the volume of IL#18 (X_1_) ranged between 0 µL and 50 µL, and the volume of EAc (X_2_) ranged between 250 µL and 1125 µL.

A coded representation of the factors was used for calculation purposes. The response under study was the total carotenoid concentration (µg/gDCW). The model used to express the responses was a second-order polynomial model, represented by Equation (1):

Y_i_ = β_0_ + β_1_X_1_ + β_2_X_2_ + β_12_X_12_ + β_11_X_1_^2^ + β_22_X_2_^2^ (1)

where Y_i_ is the experimental response from the experiment i (dependent variable), β constants are the parameters of the polynomial model, and X is the experimental factor level (independent coded units variable).

All assays were performed in 15 mL Falcon^®^ tubes to improve mixing, with 400 µL of bacterial cell suspension (35.5 gDCW/L) and variable volumes of both EAc and IL#18, according to the Doehlert distribution. The tubes were vortexed and then placed in an overhead shaker (Heidolph Reax 20, Germany) at room temperature (22 ± 2 °C). After 20 min, the tubes were collected and the mixtures were centrifuged at 15,000× *g* for 15 min (Biofuge 15 Centrifuge, Heraeus Sepatech, Germany). The upper layer containing the carotenoids was then removed and measured. Carotenoid quantification was performed through spectrophotometer analysis, as previously described, and results were evaluated in terms of total carotenoid extracted (µg/gDCW).

Fischer-statistical tests (F tests) were applied to evaluate the significance of the variances due to the effectiveness of the model fitting and the experimental error, as described by Fernandes et al. [29].

### 2.4. Validation of the Extraction Procedure with IL#18 and EAc

For the extraction using the novel methodology with IL#18 and EAc, under optimal conditions, a new lot of strain 1B biomass was produced, as described above. Wet biomass, ethyl acetate, and IL#18 were placed in 15 mL Falcon^®^ tubes under the conditions determined by #ED2. The tubes were vortexed and then placed in an overhead shaker (Heidolph Reax 20, Germany) at room temperature. After 20 min, the tubes were collected, and the mixtures were centrifuged at 15,000× *g* for 15 min (Biofuge 15 Centrifuge, Heraeus Sepatech, Germany). The upper layer containing the carotenoids was then removed and substituted with new EAc. The extraction procedure was repeated three times.

A conventional extraction procedure (with DMSO) was also performed according to Fernandes et al. [29] to compare with the optimized protocol. Prior to extraction, the biomass was dried in an oven at 55 °C to reduce the water content to 60%. Then, the biomass was mixed with DMSO and placed in an orbital incubator at 50 °C for 1 h, after which separation proceeded through centrifugation, as described above. The upper layer containing the carotenoids was removed and replaced with fresh DMSO. The process was repeated until no coloration was observed in the upper layer.

Between extractions, carotenoid samples were kept at 5 °C, protected from the light to avoid carotenoid deterioration. At the end of the extraction assays, carotenoid quantification (µg/gDCW) was performed through spectrophotometry, as previously described.

## 3. Results and Discussion

### 3.1. Extraction of Carotenoids Using ILs

#### 3.1.1. Screening Assays

**Figure 1:**
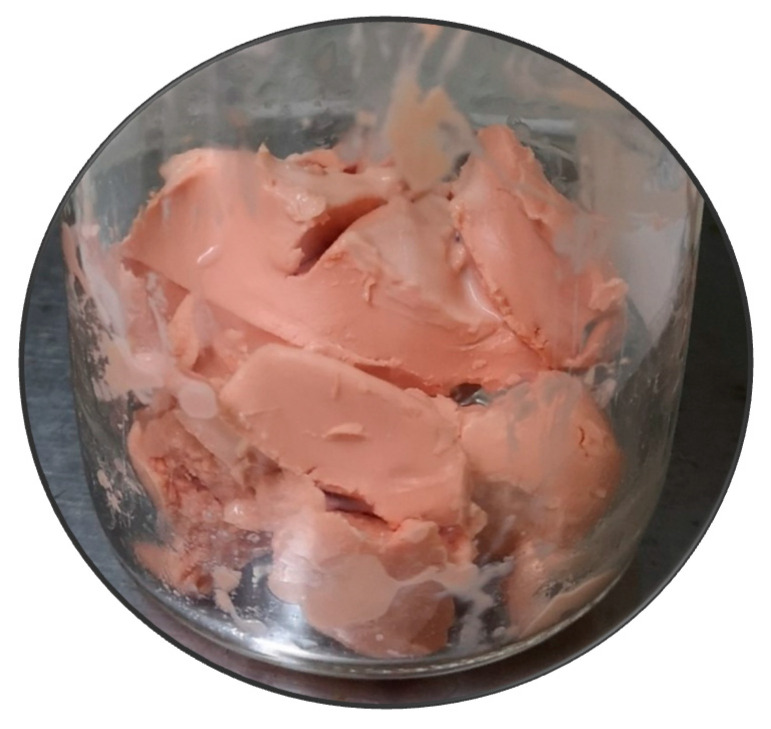
Wet biomass of *G. alkanivorans* strain 1B, cultivated in a chemostat with fructose as the C-source and sulfate as the S-source, after centrifugation.

Using the library of ILs at LNEG, nineteen different ILs were selected, following previous experiences with lignocellulosic biomass extraction, to evaluate their potential for application in carotenoid extraction from cells of *G. alkanivorans* strain 1B (Figure 1). The ILs studied in this work were selected for having sufficiently distinct either physical or chemical properties. Hence, among the properties considered to be critical in the ionic liquids’ efficiency in terms of carotenoid extraction were the following: (a) the anion influence, namely hydrophilicity and hydrophobicity and complexing ability, (b) alkyl chain length in the cation, (c) presence of functional groups, (d) Hammett acidity and basicity, and (e) viscosity and thermal stability.

For the screening, based on a protocol described by Ruiz et al. [39], a new procedure was formulated, where 400 µL of concentrated strain 1B biomass suspension was extracted with a mixture of EAc and each tested IL (#1 to #19) in a 4:3:3 ratio, respectively, by mixing it at room temperature overnight in a 1.5 mL Eppendorf tube.

**Figure 2:**
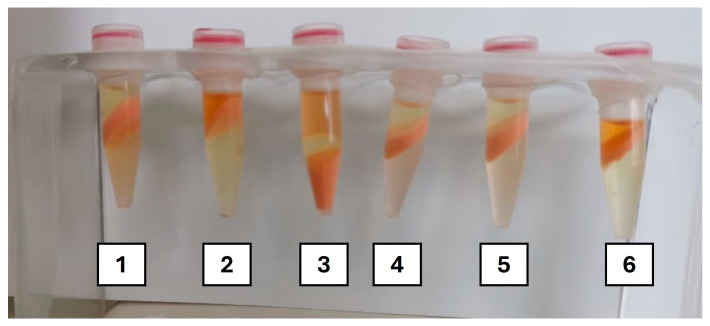
Representation of the screening of the potential of different ionic liquids (IL#1 to IL#6) toward carotenoid extraction coupled with ethyl acetate (phase-forming solvent).

After centrifugation, the results of carotenoid extraction were evaluated (visually and quantitatively), as illustrated in Figure 2. In this context, results were evaluated not just by their ability to enhance carotenoid extraction, but also the formation of a biphasic system with an isolated layer of ethyl acetate containing the carotenoids, separated from water and biomass, to facilitate analysis and recovery for future application.

**Figure 3:**
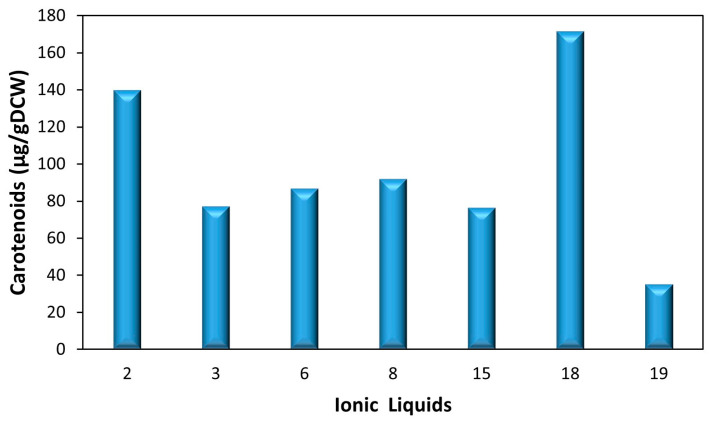
Total carotenoids (µg/gDCW), measured by spectrophotometry, after extraction with different ILs, with EAc as a co-solvent.

Initial observation revealed that out of the nineteen ILs tested (Table 1), twelve were able to form a biphasic system, clearly separating EAc and water. Of these, only seven presented visible coloration in the EAc phase, indicating significant carotenoid extraction. These seven ILs were then selected for total carotenoid quantification through a scanning spectrum, ranging the wavelengths from 380 to 900 nm [29,40]. Figure 3 shows the quantification of total carotenoids (µg/gDCW) obtained. Results demonstrate that the lowest levels of extraction were observed with IL#19 (34 µg/gDCW), followed by ILs #3, #6, #8, and #15 (between 76 and 92 µg/gDCW). The highest quantity of carotenoids was extracted when IL#2 (139 µg/gDCW) and IL#18 (171 µg/gDCW) were used combined with EAc.

These results likely emerged from the correlation of different factors. Comparing carotenoid extraction in the different imidazolium ILs suggested that extraction is favored by ILs with a cation with a shorter alkyl chain length. Shorter chains reduce the IL’s viscosity and influence its ability to dissolve in organic solvents, often resulting in better extraction efficiencies compared to longer chains due to lower viscosity and better mass transfer [42,43].

Comparing IL#18 and IL#2 with the remaining ILs, it became clear that the anion plays a significant role in carotenoid extraction. In this case, the presence of phosphates (in the form of diethyl phosphate and dimethyl phosphate) likely had a positive effect on extraction. Due to their hydrophilic nature, these phosphates improve the solubility of polar compounds, such as carotenoids, facilitating the contact between phases and leading to higher extraction efficiencies [44,45]. Furthermore, diethyl phosphate has been shown to form stable complexes with carotenoids, enhancing the extraction efficiency, while dimethyl phosphate has been shown to dissolve complex biopolymers [46,47,48].

Nonetheless, the most influential factor seems to be the presence of phosphonium anion, as IL#18 resulted in the highest extraction. Moreover, the phosphonium-based IL#3 also resulted in significant extraction, despite its relatively larger size, which could have hindered carotenoid extraction [49]. This is likely because phosphonium-based ILs have lower viscosities and better thermal stability, which enhances the extraction efficiency compared to imidazolium-based ILs [46,50]. Furthermore, phosphonium ILs can permeate and disrupt the phospholipid membrane, likely leading to enhanced cell rupture and facilitating the permeation of EAc into the cells, increasing the extraction of the carotenoids contained within [51]. However, when a phosphonium-based IL with an aromatic ring (4-methylbenzenesulfonate anion) was used (IL#7), no biphasic layer was formed with EAc and, therefore, the extraction of carotenoids could not be assessed.

Overall, this preliminary screening allowed the selection of an IL that highly increased the extraction efficiency (IL#18), while being easily recovered and reused. With this information and knowing its properties as a lipidic membrane permeabilizer that facilitates carotenoid extraction through the EAc, further optimization of the extraction process with IL#18 and EAc was performed using a response surface methodology, based on the Doehlert uniform shell design.

Additionally, alternative extraction protocols were tested with each of the 19 ILs: (a) using DMSO instead of EAc as the co-solvent, maintaining the described ratio, and (b) using an orbital shaker at 150 rpm, 50 °C, instead of the overhead shaker (with both EAc and DMSO). However, the results obtained were substantially lower, regardless of the IL selected. 

#### 3.1.2. Experimental Design (ED) for Carotenoid Extraction Optimization

To optimize the extraction process with IL#18 and EAc, a response surface methodology based on the Doehlert uniform shell design for two factors (X_1_ = IL#18 volume and X_2_ = EAc volume) was used. Two sequential experimental designs (#ED1 and #ED2), with fourteen experiments each (seven conditions in duplicate), were performed using different ranges for the factors to better understand how each factor influenced the quantity of carotenoids extracted.

##### #ED1—Response Surface

In #ED1, the volume of IL#18 (X_1_) ranged between 25 µL and 1000 µL, and the volume of EAc (X_2_) ranged between 250 µL and 2000 µL. Table 2 shows the set of tests performed within the experimental domain and the response evaluated (total carotenoids extracted, μg/gDCW). These results indicate that both factors studied influenced the response; however, this influence changed according to the conditions tested.

Tests 1–2, 3–4, and 5–6 (Table 2) showed the effect of varying the amount of IL#18 for a constant amount of EAc (1125 µL) at the center of the experimental domain. Increasing IL#18 from 25 µL to 512.5 µL (tests 7–8 and 1–2), total carotenoids extracted increased by about 44%, the highest value for this study (from 543 to 782 µg/gDCW). When the amount of IL#18 was further increased to 1000 µL (tests 3–4), the total carotenoids extracted decreased by 73% (from 782 to 210 µg/gDCW), which was lower than that using 25 µL of IL#18.

At the highest concentration of EAc tested (1882.75 µL, tests 7–8 and 13–14), increasing the concentration of IL#18 from 268.75 µL to 756.25 µL had almost no effect on carotenoid extraction, with both conditions resulting in extraction values close to the maximum (from 748 to 782 µg/gDCW, respectively). Conversely, when using the lowest concentration of EAc (367.25 µL, tests 9–10 and 11–12), a similar increase in the concentration of IL#18 resulted in a decrease of 66% in carotenoid extraction, to the lowest level of the assay (from 342 to 116 µg/gDCW).

Keeping IL#18 at 268.75 µL and increasing EAc from 367.25 µL to 1882.75 µL (tests 9–10 and 13–14) resulted in an increase of 118% in carotenoid extraction (from 342 to 748 µg/gDCW). This effect was further accentuated with a higher concentration of IL#18 (756.25 µL tests 7–8 and 11–12), where a similar increase in EAc resulted in a 557% increase in carotenoid extraction (from 116 to 763 µg/gDCW).

Simultaneously increasing IL#18 and EAc from 268.75 µL and 367.25 µL to 512.5 µL and 1125 µL, respectively, increased carotenoid extraction by 128% (from 342 to 782 µg/gDCW). Further increases of both factors to 756.25 µL and 1882.75 µL, respectively, resulted in no significant change, nor did decreasing IL#18 to 268.75 µL and increasing EAc to 1882.75 µL.

**Figure 4:**
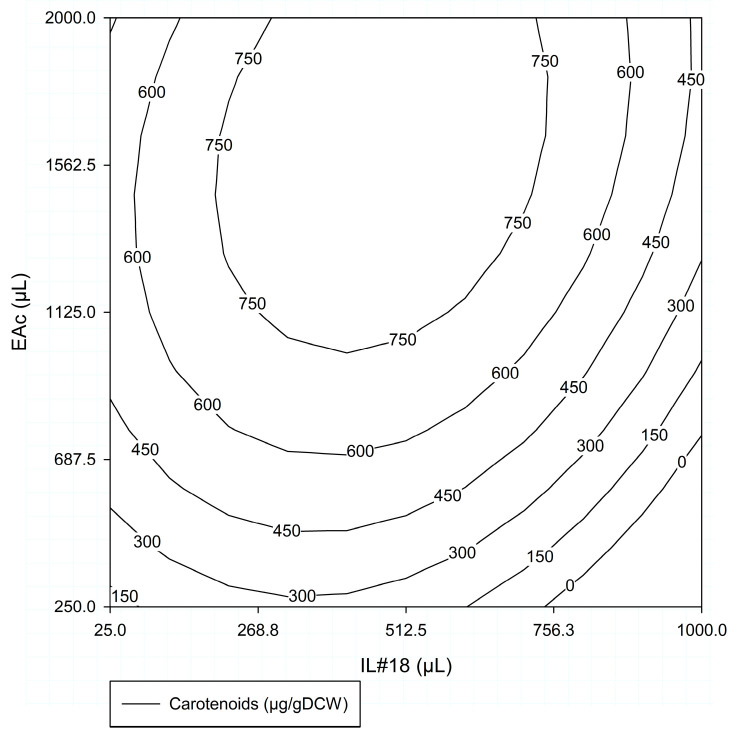
Response surface for total carotenoids extracted (µg/gDCW) obtained in #ED1, for the factors IL#18 volume (25–1000 µL) and EAc volume (250–2000 µL).

Figure 4 shows the response surface obtained for the total carotenoids extracted (µg/gDCW) within the experimental domain for the two factors studied (IL#18 volume: 25–1000 µL and EAc volume: 250–2000 µL) in #ED1. Analyzing this response surface, it became apparent that increasing IL#18 up to 512.5 µL resulted in an increase in carotenoid extraction, especially for EAc volumes greater than 700 µL. Greater increases in IL#18, particularly above 756.25 µL, led to progressively lower carotenoid extraction. In terms of EAc, it was the most influential factor up to 1000–1125 µL, having a clear positive influence on the response. Further increases appeared to have lesser influence, as shown by the more vertical lines, and could even result in slightly lower carotenoid extraction for values above 1500 µL, depending on the amount of IL#18.

Analysis for #ED1 Factors

The data resulting from the #ED1 were further used for a regression analysis, as described by Silva et al. [37], and Table 3 summarizes the polynomial model-derived parameters.

Considering the #ED1 parameters (Table 3), it became clear that both factors tested had a significant influence on the quantity of carotenoids extracted, albeit with opposite effects. β1 corresponded to a negative value, indicating that increases in IL#18 from the central point resulted in reductions in the response, while β2 presented a positive value, indicating greater responses with increases in EAc. Furthermore, this also showed that, within the experimental domain, EAc had a greater influence than the volume of IL#18, since β2 was more than twice the absolute value of β1. β12 indicated that by increasing both IL and EAc, there was an attenuation of the negative effect caused by IL#18.

The F test showed that the variance was significantly accounted for by the values of the parameters at F (5,8), with a significance level of approximately 0.001 (Table 3). This indicates that the model represented the data adequately. A second F test was performed to determine whether the origin of the variance was due to the experimental error. The source of the variance contained was explained by the experimental error at the F (1,7) significance level of 0.05 (Table 3). Both analyses allowed to assert that the use of IL#18 in combination with EAc enhanced the carotenoid extraction process.

Overall, this preliminary ED demonstrated that (a) the combination of EAc and IL#18 increased carotenoid extraction from strain 1B biomass, (b) the optimum value of IL#18 volume should be below 512.5 µL, and (c) the maximum value of carotenoid extraction could be observed with EAc ≥ 1000 µL. However, these results also indicated that the experimental domain tested was likely excessively broad, making it difficult to correctly determine optimum conditions. Therefore, considering that one of the major setbacks of ILs and organic solvents is their cost, it became necessary to assess the possibility of reducing their concentration without significantly impacting carotenoid extraction.

##### #ED2—Response Surface

Bearing in mind the previous results and to assess the possibilty of using substantially smaller amounts of the reagents (IL#18, EAc) without losing much extraction capacity, the next aproach was to perform a new experimental design (#ED2) with lower quantities of both solvents (IL#18 volume (X_1_): 0–50 µL; EAc volume (X_2_): 250–1125 µL) while maintaining the amount of biomass. Table 4 shows the set of tests performed within the #ED2 and the response evaluated (total carotenoids extracted, µg/gDCW).

Tests 1–2, 3–4, and 5–6 (Table 4) showed the effect of varying the volume of IL#18 for a constant volume of EAc at the center of the experimental domain (687.5 µL). Increasing the IL#18 volume from 0 to 25 µL resulted in a 48% increase in total carotenoids extracted (from 310 to 458 µg/gDCW). A further increase in the volume of IL#18 to 50 µL resulted in an additional 14% increase in total carotenoids extracted (from 458 to 521 µg/gDCW). In tests 7–8 and 13–14, and 9–10 and 11–12, with EAc volumes of 1066.375 µL and 308.625 µL, respectively, increasing the IL#18 volume from 12.5 µL to 37.5 µL resulted in a small but positive increase in carotenoid extraction of 7% and 9%, respectively. Keeping IL#18 at 12.5 µL or 37.5 µL and increasing EAc from 308.625 µL to 1066.375 µL (tests 9–10 and 13–14, and 7–8 and 11–12, respectively) resulted in approximately a 45-fold increase in total carotenoid extraction in both cases (from 15.4 to 696 µg/gDCW and from 16.7 to 748 µg/gDCW, respectively). Increasing both IL#18 and EAc from 12.5 µL and 308.625 µL to 25 µL and 687.5 µL (tests 9–10 and 1–2, respectively) resulted in a 30-fold increase (from 15.4 to 458 µg/gDCW), while a further increase to 37.5 µL and 1066.375 µL resulted in a 63% increase to 748 µg/gDCW (tests 7–8).

**Figure 5:**
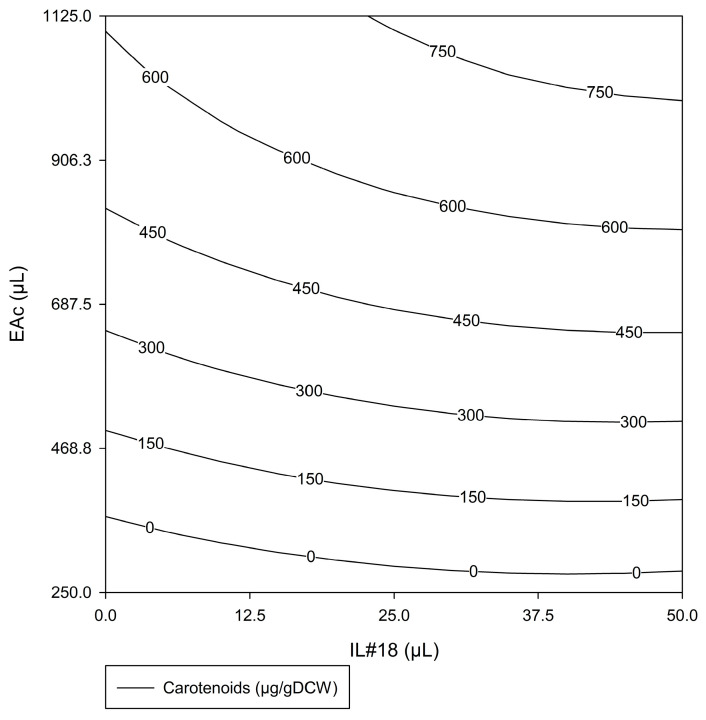
Response surface for total carotenoids extracted (µg/gDCW) obtained in #ED2, for the factors IL#18 volume (0–50 µL) and EAc volume (250–1125 µL).

Once again, #ED2 demonstrated the influence of both factors for bacterial carotenoid extraction. Figure 5 shows the response surface obtained for the total carotenoids extracted (µg/gDCW) within the experimental domain tested in #ED2 (IL#18 volume: 0–50 µL; EAc volume: 250–1125 µL). Analyzing the response surface, it was possible to observe that both factors had a positive influence. Increasing either factor individually increased the response. However, EAc volume was clearly more influential, especially in the lower quadrants, corresponding to lower volumes of EAc, as indicated by the almost horizontal lines. With greater volumes of EAc, IL#18 became more influent, as demonstrated by the increase in slope in the upper quadrants.

Overall, these results indicate that better extraction conditions can be achieved with ≥25 µL of IL#18 in combination with ≥1000 µL of EAc, with an optimal condition at 50 µL of IL#18 with 1125 µL of EAc, to guarantee a maximum value of extracted carotenoids.

Analysis for #ED2 Factors

The data obtained from the #ED2 were further used for a regression analysis, and the polynomial-model-derived parameters (β0–β22) are shown in Table 5. Considering the #ED2 parameters (Table 5), in contrast to #ED1, both factors had a positive influence on the response, as indicated by the positive values of both β1 and β2. Nevertheless, similar to #ED1, EAc was substantially more influential for carotenoid extraction, with β2 presenting a value more than 5-fold higher than β1. The F tests were repeated for the second model. F (5,8) showed a significance level of approximately >0.001, reinforcing the adequacy of the model to reproduce the experimental data, and the source of the variance contained was explained by the experimental error at the F (1,7) significance level of 0.01 (Table 5).

Observing the combined results for #ED1 (Figure 4) and #ED2 (Figure 5), it was evident that for lower EAc volumes, the amount of EAc was the limiting factor. A small volume of EAc can be easily saturated with carotenoids, making it difficult to increase extraction, regardless of the carotenoids’ ease of extraction. With higher volumes of EAc, this no longer occurred, and the amount of IL#18 gained importance, as the limiting factor became the access to the carotenoids contained within the cell. In this circumstance, increasing the IL#18 volume likely led to increased cell rupture, resulting in a greater quantity of carotenoids being released from the cell and, therefore, becoming available for extraction.

It is also important to remember that the 400 µL of biomass (cell suspension with 35.5 gDCW/L) used in every extraction assay was composed of more than 90% water, which acted as a co-solvent, influencing the interactions between biomass, IL#18, and EAc. For lower IL#18 volumes, particularly in #ED2, the excess water could affect the supramolecular structure of the IL, weakening the effects of IL#18, both on the membrane rupture and carotenoid solubilization [52]. Additionally, for lower amounts of EAc, a high water content, combined with an increased net biomass concentration and the hydrophobic properties of strain 1B, will likely lead to the formation of strong emulsions between biomass, EAc, and water, which, as previously reported [37], will hinder proper mixing of the solvents and biomass, lowering the extraction results. Any excessive increase in one solvent in relation to the other could hinder access to the biomass containing the carotenoids, thus negatively affecting extraction.

Comparing the results obtained in #ED1 (Table 2) and #ED2 (Table 4), it was evident that the highest carotenoid extraction was similar in both cases, though slightly higher in #ED1. Furthermore, comparing the optimal extraction conditions predicted from the #ED2 response surface (50 µL of IL#18 with 1125 µL of EAc, for 400 µL of biomass; Figure 5) with the conditions that allowed maximum carotenoid extraction in #ED1 (512.5 µL of IL#18 with 1125 µL of EAc, for 400 µL of biomass; Table 2: tests 1–2), it became clear that a similar extraction result (>750 mg/gDCW) could be obtained with 90% less IL#18, without changing any other factor. Therefore, the condition selected for high carotenoid extraction from biomass of *G. alkanivorans* strain 1B (400 mL of a cell suspension with 35.5 gDCW/L) was 50 µL of IL#18 with 1125 µL of EAc, as this allowed a reduction in the use of IL without compromising carotenoid extraction.

### 3.2. Validation of the Extraction Procedure with IL#18 and EAc

To compare the new methodology using IL#18 and EAc toward bacterial carotenoid extraction with the previously established protocol using DMSO [28,29], a new assay was conducted using strain 1B biomass. For this, a new batch of strain 1B biomass was prepared under the same conditions (growth in a chemostat with 20 g/L of fructose and concentrated biomass suspension adjusted to the same DCW (≈36 g/L)) and subjected to the two different extraction protocols. In this case, to ensure proper comparison between procedures, both extraction protocols were performed with three sequential extractions to ensure maximal carotenoid extraction.

**Figure 6:**
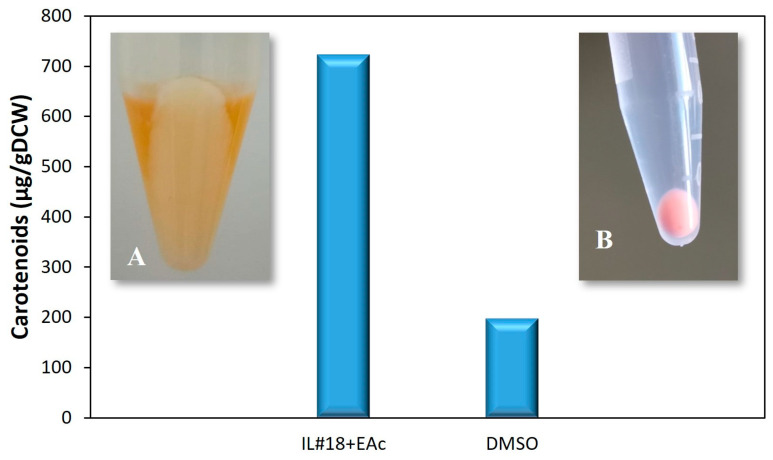
Total carotenoids (µg/gDCW), measured by spectrophotometry, after extraction with 50 µL of IL#18 with 1125 µL of EAc (optimized conditions) and the conventional extraction protocol using DMSO. Discoloration of *G. alkanivorans* strain 1B biomass after three sequential extractions using: (**A**) 50 µL of IL#18 with 1125 µL of EAc versus (**B**) DMSO.

The results obtained in these assays are presented in Figure 6 and Figure S1 (Supplementary Materials). As expected, these results confirmed the effectiveness of the new extraction methodology developed in this study over the traditional extraction procedure with DMSO. Indeed, Figure 6 shows a 264% increase in carotenoid extraction when comparing the DMSO extraction protocol with the novel procedure with IL#18 and EAc, from 199 to 724 μg/gDCW, respectively. Moreover, after three sequential extractions, the final extracted biomasses, represented in the same figure, also demonstrated clear differences. The biomass extracted with IL#18 and EAc (Figure 6A) became almost white, indicating a complete carotenoid extraction, while the biomass extracted with DMSO still maintained a pink coloration, indicative of remaining carotenoids, even after three extraction rounds, after which DMSO was incapable of further carotenoid extraction. Additionally, Figure S1 demonstrates that the extracts obtained with both extraction methods presented absorbance spectra associated with carotenoids, similar to previous works [28,29,40]. This indicates that the leftover carotenoids in the biomass extracted with DMSO were significant, and that the increase in carotenoid extraction with IL#18 and EAc was the result of a more complete recovery of carotenoids from bacterial biomass. This suggests that previous results obtained with the DMSO extraction procedure [28,29,30] likely substantially undervalued carotenoid production by strain 1B.

In addition, it is important to highlight that the value obtained for carotenoid extraction using the novel method (724 μg/gDCW) was slightly lower than that predicted through #ED2. This was likely the result of using a different biomass batch, since despite culture conditions being kept constant, the light source was not controlled. Since light intensity can greatly influence carotenoid production, even with similar culture conditions [28], some variation is expected in the overall carotenoid content between biomass batches. Nevertheless, the result obtained was within the experimental range observed for both EDs, validating the methodology established using IL#18 with EAc as a co-solvent.

Lastly, comparing both methods, the traditional approach requires an oven-drying pretreatment step to reduce water content in the biomass from ≈90% to ≈60%, and demands an extraction temperature of 50 °C, both of which are energy-intensive. In contrast, the novel approach using IL#18 and EAc at room temperature can achieve substantially greater efficiency without any pretreatment. This makes the extraction process simpler and potentially more energy-efficient.

As previously stated, microbial carotenoid extraction is an area of increasing interest and, therefore, different approaches have been pursued to achieve better and more efficient extraction. Beyond ILs, the use of deep eutectic solvents (DESs), supercritical fluids (SCFs), and enzyme-assisted extraction (EAE), amongst many others, has been studied by different researchers [53,54,55,56,57,58]. However, when concerning ILs and DESs, their applications to microorganisms have mostly been centered on carotenoid extraction of microalgae and yeast [11,13,57]. In fact, to the best of our knowledge, the present work is the first application of ILs to extract carotenoids from bacterial biomass.

Considering some of the most common examples of microbial carotenoid extraction with ILs, they are typically very effective at extracting substantial amounts of carotenoids, but either use comparatively greater amounts of IL, greater temperatures, or greater contact times. An example of this is the research on the extraction of β-carotene from the yeast *Rhodotorula glutinis* using ammonium-based ILs at room temperature. The extraction process demonstrated efficiencies of up to 90% but required a 30% IL concentration [54]. Another example is the use of imidazolium-based IL/water mixtures at 30 °C to extract astaxanthin from the microalga *Haematococcus pluvialis.* It effectively permeabilized the cyst cells and allowed almost complete astaxanthin recoveries (>99%), along with moderate lipid extractions (∼82%), with 6.7% (*v*/*v*) IL in water solution but taking 60 min of contact time [55].

DESs have emerged as sustainable alternatives for carotenoid extraction, offering low toxicity and high solvation power, despite potentially greater viscosity. However, similar to ILs, there are very few works regarding their application toward carotenoid extraction in bacteria. One of the few existing works focused on the obtention of astaxanthin-rich extracts from the Gram-negative bacterium *Paracoccus carotinifaciens* using a choline chloride:acetic acid (CC-C2) DES, and reported a notable extraction yield increase when compared to the conventional method used [59]. However, this extraction required the combination with ultrasounds and a temperature of 65 °C.

Even though comparison between results obtained with different methods on different microorganisms is difficult, it becomes clear that there are very diverse approaches to increase carotenoid extraction. In this study, the method developed for the bacterium *G. alkanivorans* strain 1B has the advantage of working on wet biomass, at room temperature, without intense pressures or prolonged extraction times, and using comparatively small amounts of IL. Furthermore, it drastically increased the extraction yield on a strain known to be difficult to extract, without the need for any further pretreatment. To properly determine if other methods could yield similar results, these would have to be applied to the biomass of strain 1B, but considering the remarkable increase observed in carotenoid extraction using IL#18 + EAc it is unlikely that the results would be substantially better.

In addition, strain 1B is known for its potential to be used in biodesulfurization, a process that is highly dependent on substantial amounts of active microbial biomass, and that consequently generates substantial amounts of exhausted biomass residues. The novel extraction process could help to increase the extraction yields and thus increase the carotenoid production associated with the biodesulfurization process, thus helping the process become more economically sustainable [60,61].

## 4. Conclusions

The bacterium *G. alkanivorans* strain 1B is a hyper-pigment producer; however, its natural adaptations to hydrophobic environments make extraction of the high-added-value compounds particularly difficult. Previous works demonstrated that conventional extraction methods were not effective, since cells would retain most coloration even after several extraction steps. Therefore, this study explored the option of complementing solvent extraction of carotenoids with ILs to facilitate cell rupture and increase extraction yields. An initial screening showed that, when combined with EAc as a co-solvent, both imidazolium- and phosphonium-based ILs had high extraction potential of strain 1B carotenoids. However, the greatest extraction result was obtained using a phosphonium-based IL with a diethyl phosphate anion (IL#18: tributyl(ethyl)phosphonium diethyl phosphate). After optimization through response surface methodology (#ED1 and #ED2), a new extraction procedure was developed using 50 µL of IL#18 with 1125 µL of EAc, for 400 µL of bacterial biomass (cell suspension ≈ 36 gDCW/L). This method increased carotenoid extraction by 264% compared to the traditional DMSO extraction. This novel methodology not only drastically increased the extraction efficiency but also allowed the direct utilization of wet biomass (i.e., concentrated cell suspension), eliminating the need for a drying step and lowering the extraction temperature from 50 °C to room temperature. Both changes are fundamental when developing a process aimed at future industrial application, as drying and heating are energy-intensive steps that heavily contribute to net process costs and emissions. Hence, this novel methodology, using an IL to promote enhanced carotenoid extraction from bacterial biomass, appears as a promising eco-friendly alternative tool, contributing to making carotenoid extraction more efficient and sustainable. Future studies will be necessary to optimize the subsequent purification and concentration steps, to properly assess the recuperation and reutilization of EAc and IL#18, and to establish purification procedures for the carotenoid mix, as well as test the procedure with pigmented biomasses of different complexities (e.g., Gram-positive versus Gram-negative bacteria and different bacterial genera/species).

## Figures and Tables

**Table 1:** List of ionic liquids tested and their respective brands.

No. (#)	Ionic Liquids	Brand
1	1-Ethyl-3-methylimidazolium bis(trifluoromethylsulfonyl)imide	Solchemar
2	1,3-Dimethylimidazolium dimethyl phosphate	Io-li-tec
3	Trihexyl(tetradecyl)phosphonium bis(trifluoromethylsulfonyl)imide	Solchemar
4	1-Butyl-3-methylimidazolium bis(trifluoromethylsulfonyl)imide	Io-li-tec
5	1-Hexyl-3-methylimidazolium bis(trifluoromethylsulfonyl)imide	Io-li-tec
6	1-Ethyl-3-Methylimidazolium hydrogensulfate	Solchemar
7	Triisobutyl(methyl)phosphonium tosylate	Io-li-tec
8	1-Ethyl-3-Methylimidazolium thiocyanate	Io-li-tec
9	1-Butyl-3-Methylimidazolium thiocyanate	Io-li-tec
10	1-Butyl-3-Methylimidazolium tetracholoroferrate (III)	Io-li-tec
11	1-Butyl-3-Methylimidazolium dicyanamide	Io-li-tec
12	1-Butyl-3-Methylimidazolium trifluoromethanesulfonate	Io-li-tec
13	1-Ethyl-3-Methylimidazolium dihydrogen phosphate	Solchemar
14	1-Ethyl-3-methylimidazolium trifluoromethanesulfonate	Solchemar
15	1-Ethyl-3-Methylimidazolium methanesulfonate	Solchemar
16	Didecyl-dimethylammonium nitrate	Io-li-tec
17	2-hydroxyethylammonium formate	Io-li-tec
18	Tributyl(ethyl)phosphonium diethyl phosphate	Solchemar
19	1-Methylimidazolium bis(trifluoromethylsulfonyl)imide	Io-li-tec

**Table 2:** Tests for #ED1 according to a Doehlert distribution for two factors: IL#18 volume (25–1000 µL) and EAc volume (250–2000 µL), and the response evaluated (total carotenoids, µg/gDCW).

Test (#)	Factors			Response
	IL#18 Volume (25–1000 µL)	EAc Volume (250–2000 µL)		Total Carotenoids (µg/gDCW)
1	512.5	1125		839.1
2	512.5	1125		725.3
3	1000	1125		210.4
4	1000	1125		210.4
5	25	1125		558.1
6	25	1125		527.8
7	756.25	1882.75		717.2
8	756.25	1882.75		808.1
9	268.75	367.25		350.9
10	268.75	367.25		333.8
11	756.25	367.25		114.3
12	756.25	367.25		117.9
13	268.75	1882.75		666.7
14	268.75	1882.75		828.3

**Table 3:** Parameters of the polynomial models representing the studied response (total carotenoids extracted) in #ED1, for the IL#18 volume (25–1000 µL) and EAc volume (250–2000 µL).

Model	Response: Total Carotenoids
Model parameters	
β0	782.3
β1	−146.05
β2	303.61
β12	139.38
β11	−405.61
β22	−251.68
Model validation (Fischer test)	
Effectiveness of the parameters	51.01
Significance level (α) F (5,8)	0.001
Lack of fit	1.4
Significance level (α) F (1,7)	<0.05
R^2^	0.97

Note: β0—response at the center of the experimental domain; β1 and β2—parameters of the factors 1 (volume of IL#18, µL) and 2 (volume of EAc, µL), respectively; β12—parameter of the interaction of the factors 1 and 2; β11 and β22—self-interaction parameters of the factors 1 and 2, respectively.

**Table 4:** Tests for #ED2 according to a Doehlert distribution for two factors: volume of IL#18 (0–50 µL) and volume of EAc (250–1125 µL), and the response evaluated (total carotenoids, µg/gDCW).

Test (#)	Factors			Response
	IL#18 Volume (0–50 µL)	EAc Volume (250–1125 µL)		Total Carotenoids (µg/gDCW)
1	25	687.5		464.9
2	25	687.5		451.2
3	50	687.5		487.6
4	50	687.5		555.0
5	0	687.5		315.7
6	0	687.5		304.5
7	37.5	1066.375		724.6
8	37.5	1066.375		770.4
9	12.5	308.625		15.4
10	12.5	308.625		15.4
11	37.5	308.625		16.7
12	37.5	308.625		16.7
13	12.5	1066.375		697.7
14	12.5	1066.375		694.3

**Table 5:** Parameters of the polynomial models representing the studied response (total carotenoids extracted) in #ED2, for the volume of IL#18 (0–50 µL) and volume of EAc (250–1125 µL).

Model	Response: Total Carotenoids
Model parameters	
β0	458.11
β1	79.21
β2	407.44
β12	28.98
β11	−42.41
β22	−104.85
Model validation (Fischer test)	
Effectiveness of the parameters	141.96
Significance level (α) F (5,8)	0.001
Lack of fit	16.79
Significance level (α) F (1,7)	0.01
R^2^	0.99

Note: β0—response at the center of the experimental domain; β1 and β2—parameters of the factors 1 (volume of IL#18, µL) and 2 (volume of EAc, µL), respectively; β12—parameter of the interaction of the factors 1 and 2; β11 and β22—self-interaction parameters of the factors 1 and 2, respectively.

## Data Availability

The authors confirm that the datasets supporting the findings and conclusions of this study are available within the article.

## Supplementary Materials

The following supporting information can be downloaded at: https://www.mdpi.com/article/10.3390/molecules29174132/s1, Figure S1: Absorbance spectra for the carotenoid extracts from *G. alkanivorans* strain 1 B biomass extracted using the DMSO extraction protocol versus the novel procedure with IL#18 and EAc.; Table S1: List of the 19 ionic liquids tested with the respective chemical structures.
