# Supplementary material for: Ionic Liquids toward Enhanced Carotenoid Extraction from Bacterial Biomass
Source: Molecules. 2024 Aug 30;29(17):4132. doi: 10.3390/molecules29174132 (PMC11397713; doi:10.3390/molecules29174132)
Supplement: Supplementary file 1 [file molecules-29-04132-s001.zip › molecules-3145607-supplementary.pdf]

Table S1. List of the 19 ionic liquids tested with the respective chemical structures.

Nr (#)	Ionic Liquids
**1**	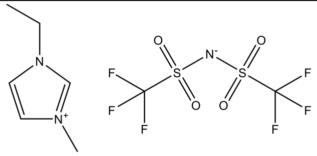 1-Ethyl-3-methylimidazolium bis(trifluoromethylsulfonyl)imide
**2**	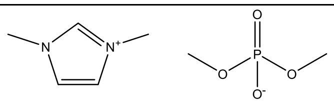 1,3-Dimethylimidazolium dimethyl phosphate
**3**	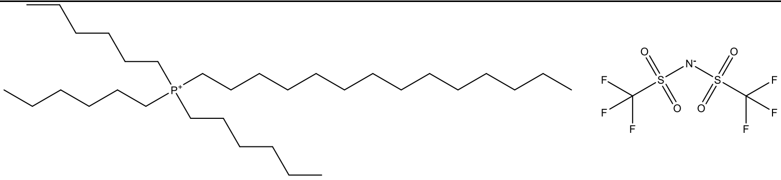 Trihexyl(tetradecyl)phosphonium bis(trifluoromethylsulfonyl)imide
**4**	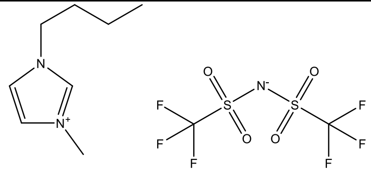 1-Butyl-3-methylimidazolium bis(trifluoromethylsulfonyl)imide
**5**	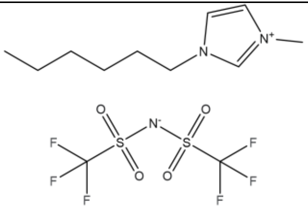 1-Hexyl-3-methylimidazolium bis(trifluoromethylsulfonyl)imide
**6**	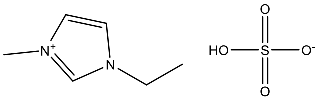 1-Ethyl-3-Methylimidazolium hydrogensulfate
**7**	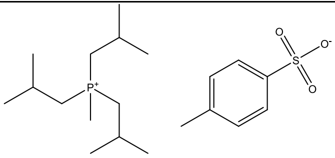 Triisobutyl(methyl)phosphonium tosylate

**8**	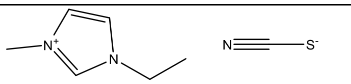 1-Ethyl-3-Methylimidazolium thiocyanate
**9**	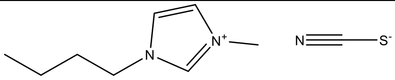 1-Butyl-3-Methylimidazolium thiocyanate
**10**	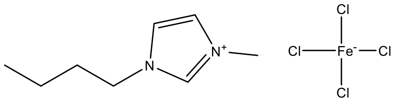 1-Butyl-3-Methylimidazolium tetracholoroferrate (III)
**11**	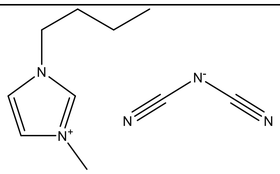 1-Butyl-3-Methylimidazolium dicyanamide
**12**	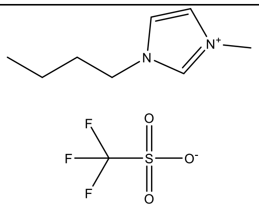 1-Butyl-3-Methylimidazolium trifluoromethanesulfonate
**13**	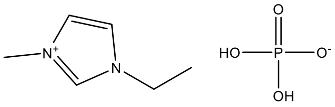 1-Ethyl-3-Methylimidazolium dihydrogen phosphate
**14**	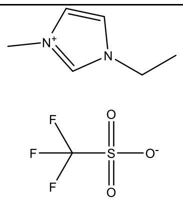 1-Ethyl-3-methylimidazolium trifluoromethanesulfonate
**15**	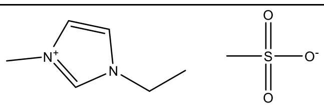 1-Ethyl-3-Methylimidazolium methanesulfonate
**16**	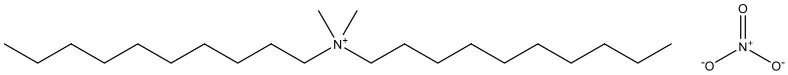 Didecyl-dimethylammonium nitrate

**17**	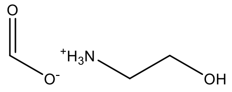 2-hydroxyethylammonium formate
**18**	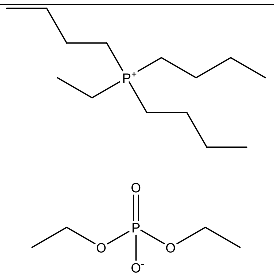 Tributyl(ethyl)phosphonium diethyl phosphate
**19**	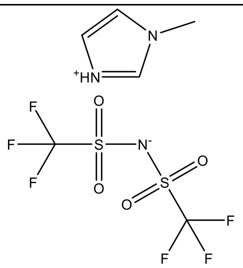 1-Methylimidazolium bis(trifluoromethylsulfonyl)imide

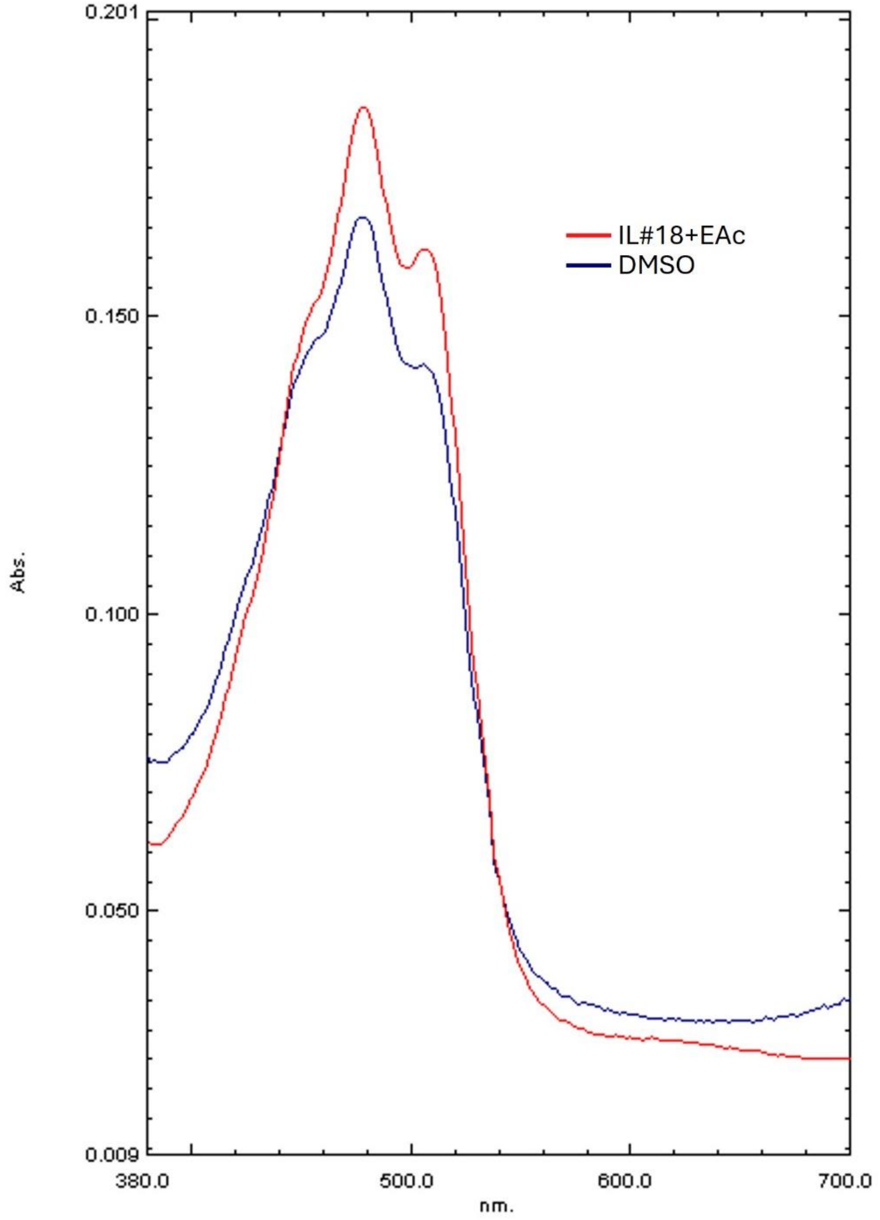

Figure S1. Absorbance spectra for the carotenoid extracts from *G. alkanivorans* strain 1 B biomass extracted using the DMSO extraction protocol *versus* the novel procedure with IL#18 and EAc.
